# Extraction Methods and Characterization of β-Glucans from Yeast Lees of Wines Produced Using Different Technologies

**DOI:** 10.3390/foods13243982

**Published:** 2024-12-10

**Authors:** Ana Chioru, Aurica Chirsanova, Adriana Dabija, Ionuț Avrămia, Alina Boiştean, Ancuța Chetrariu

**Affiliations:** 1 Faculty of Food Technology, Technical University of Moldova, MD-2004 Chișinău, Moldova; aurica.chirsanova@toap.utm.md (A.C.); alina.boistean@toap.utm.md (A.B.); 2 Faculty of Food Engineering, Stefan cel Mare University of Suceava, 720229 Suceava, Romania; adriana.dabija@fia.usv.ro (A.D.); ionut.avramia@usm.ro (I.A.)

**Keywords:** winery yeast lees, β-glucans, ultrasound, autolysis, alkali–acidic extraction

## Abstract

Wine lees, the second most significant by-product of winemaking after grape pomace, have received relatively little attention regarding their potential for valorization. Despite their rich content in bioactive components such as β-glucans, industrial utilization faces challenges, particularly due to variability in their composition. This inconsistency impacts the reliability and standardization of final products, limiting broader adoption in industrial applications. β-Glucans are dietary fibers or polysaccharides renowned for their diverse bioactive properties, including immunomodulatory, antioxidant, anti-inflammatory, antitumor, and cholesterol- and glucose-lowering effects. They modulate the immune system by activating Dectin-1 and TLR receptors on immune cells, enhancing phagocytosis, cytokine production, and adaptive immune responses. Their antioxidant activity arises from neutralizing free radicals and reducing oxidative stress, thereby protecting cells and tissues. β-Glucans also exhibit antitumor effects by inhibiting cancer cell growth, inducing apoptosis, and preventing angiogenesis, the formation of new blood vessels essential for tumor development. Additionally, they lower cholesterol and glucose levels by forming a viscous gel in the intestine, which reduces lipid and carbohydrate absorption, improving metabolic health. The biological activity of β-glucans varies with their molecular weight and source, further highlighting their versatility and functional potential. This study investigates how grape variety, vinification technology and extraction methods affect the yield and properties of β-glucans extracted from wine lees. The physico-chemical and mineral composition of different wine lees were analyzed, and two extraction methods of β-glucans from wine lees were tested: acid-base extraction and autolysis. These two methods were also tested under ultrasound-assisted conditions at different frequencies, as well as without the use of ultrasound. The β-glucan yield and properties were evaluated under different conditions. FTIR spectroscopy was used to assess the functional groups and structural characteristics of the β-glucans extracted from the wine lees, helping to confirm their composition and quality. Rheological behavior of the extracted β-glucans was also assessed to understand the impact of extraction method and raw material origin. The findings highlight that vinification technology significantly affects the composition of wine lees, while both the extraction method and yeast origin influence the yield and type of β-glucans obtained. The autolysis method provided higher β-glucan yields (18.95 ± 0.49% to 39.36 ± 0.19%) compared to the acid–base method (3.47 ± 0.66% to 19.76 ± 0.58%). FTIR spectroscopy revealed that the β-glucan extracts contain a variety of glucan and polysaccharide types, with distinct β-glucans (β-1,4, β-1,3, and β-1,6) identified through specific absorption peaks. The rheological behavior of suspensions exhibited pseudoplastic or shear-thinning behavior, where viscosity decreased significantly as shear rate increased. This behavior, observed across all β-glucan extracts, is typical of polymer-containing suspensions. These insights are critical for optimizing β-glucan extraction processes, supporting sustainability efforts and waste valorization in the wine industry. Efficient extraction of β-glucans from natural sources like wine lees offers a promising path toward their industrial application as valuable functional compounds.

## 1. Introduction

In an era where we produce a larger amount of waste products, their valorization, in the context of the circular economy, is not only an opportunity but a pressing necessity [1,2,3]. This study investigates the physicochemical and mineral composition of winery yeast lees, compares two distinct methods for extracting β-glucans, and evaluates the quality of the resulting extracts through FTIR spectroscopy. Furthermore, the study analyzes the rheological properties of β-glucan suspensions, providing insights into their potential applications in various industries, including food, pharmaceuticals, and cosmetics. These findings aim to contribute to the development of sustainable solutions for waste valorization, supporting the transition to a circular economy. Winemaking is a sector of the economy that generates by-products containing numerous valuable compounds, many of which are still understudied and underutilized [4,5]. According to the report of the International Organization of Vine and Wine from April 2024 [6], worldwide in 2023, 237 mhL of wine were produced. Of the total amount of wine produced, approximately 2–6% represents wine lees [7], which would be approximately 4,740,000–14,220,000 hL produced worldwide in 2023. One of the valuable compounds of this by-product are β-glucans, a polysaccharide with interesting physical-chemical, functional and therapeutic properties. Obtaining β-glucans from yeasts has been quite studied in recent years, yeasts being a rich source of β-glucans, which represent 55–65% of their cell wall [8]. Many authors have tested different methods of extracting β-glucans from dry or grown yeast *Saccharomyces cerevisiae* (β 1-3 and β 1-6 glucans) [9,10,11,12]. In recent years, residual yeasts have increasingly been studied as a source of β-glucans, especially brewer’s yeasts [13,14,15,16]. The extraction of β-glucans from residual winemaking yeasts is much more difficult because they contain tartaric salts, pectin, and phenolic compounds [17]. These have already been studied [8,18].

The winemaking industry constitutes one of the pillars of the economy of the Republic of Moldova, providing contributions to the generation of employment in rural areas and to the country’s exports with a production scale of approximately 17.1 mdaL in 2021, despite the global and regional challenge [19]. Taking into account that 2–6% of the amount of wine produced will be residual yeasts, then, in 2021, approximately 342–1026 thousand daL was produced in the Republic of Moldova [7,20]. The sustainable management of by-products from winemaking, particularly wine lees, is gaining increasing attention. Despite their potential, wine lees remain an underutilized resource, often discarded or employed in limited applications such as ethanol production and soil fertilizers. However, these applications are more a way to reduce disposal costs than a real by-product valorization strategy [21].

In this study, we compared two methods for extracting β-glucans from winery yeast lees derived from four wine types produced using different technologies. The first method involves alkaline–acid extraction with ultrasound, using NaOH and acetic acid, while the second employs ultrasound-assisted autolysis followed by acid extraction with acetic acid. These two methods were selected due to their distinct advantages. The acid–base method effectively solubilizes the structural components of the yeast cell wall and breaks the bonds between polysaccharides and proteins, thereby releasing β-glucans. This method is also cost-efficient, utilizes readily available reagents, and can be easily scaled up for industrial applications. The autolysis extraction method promotes the degradation of the yeast cell wall through natural enzymatic mechanisms, making it eco-friendly and sustainable. Each approach has benefits and limitations. For instance, other types of extractions, like solvent extraction techniques, can produce great efficiency but present safety and environmental issues; enzymatic techniques are cost-effective and ecologically friendly; and supercritical fluid extraction is novel but necessitates expensive equipment. Most yeast samples studied originated from wine storage, where yeast cells undergo autolysis or enter inactive phases, leading to thinner cell walls and potentially increasing nutrient availability for active cells [22]. The aim of this study is to explore innovative methods for transforming waste into valuable resources, focusing on the winery yeast lees biomass generated in industrial processes.

## 2. Materials and Methods

### 2.1. Winery Yeast Lees

In this study, winery yeast lees from four types of wines from three Moldavian wineries were used. The four types of wine lees from three winemakers in Moldova have been summarized in Table 1.

### 2.2. Analysis of Winery Yeast Lees

#### 2.2.1. Extraction and Analysis of β-Glucans

- Ultrasound-assisted alkali–acidic extraction of β-glucans

The extraction method is the one described by Karslioglu et al., 2021 [9] with some modifications. A 25 mL volume of residual yeast biomass, of each type, was taken for the extraction. In order to remove the remaining alcohol present in the yeast biomass, it was centrifuged at 5000 rpm for 15 min. The supernatant was removed. To the solid biomass of residual yeast was added 1 M, 1.5 M and 2 M sodium hydroxide solution, in the volume ratio of 1:4. The mixture was treated with ultrasound in an ultrasound water bath at 80 °C, applying ultrasonic waves at 40 kHz for 15 min. It was placed in the water bath for 1 h at a temperature of 90 °C. After cooling, the suspension was centrifuged at 5000 rpm, 4 °C for 15 min. The precipitate was washed with distilled water and centrifuged at 5000 rpm, for 5 min. The supernatant was removed, and 3% acetic acid was added in the volume ratio of 1:4. The suspension was heated to 85 °C and left for 1 h in the water bath. It was left to cool and centrifuged at 5000 rpm, 4 °C for 15 min. The precipitate was washed with distilled water and centrifuged at 5000 rpm for 5 min. The supernatant was removed, and the precipitate was washed with ethanol and centrifuged at 5000 rpm, 4 °C for 15 min. Then, once again, the precipitate was washed with distilled water and centrifuged at 5000 rpm for 5 min. The obtained β-glucan compounds were dried in an air oven at 55 °C for 6 h. All chemicals used in this paper were of analytical grade and were purchased from Sigma Aldrich (St. Louis, MO, USA) and Merck, Darmstadt, Germany.

B. Ultrasound-assisted autolysis extraction of β-glucans

The same amount of yeast biomass of 25 mL was taken from each type, and it was centrifuged at 5000 rpm for 15 min. Sodium phosphate buffer solution (30%, pH = 7.3) was added to the yeast biomass. The mixture was incubated for 24 h at 55 °C. Then, it was centrifuged at 5000 rpm, 4 °C for 15 min. The precipitate was washed with distilled water and centrifuged at 5000 rpm for 5 min. The supernatant was removed, and 3% acetic acid was added in the volume ratio of 1:4. The suspension was heated to 85 °C and left for 1 h in the water bath. It was left to cool and centrifuged at 5000 rpm, 4 °C for 15 min. The precipitate was washed with distilled water and centrifuged at 5000 rpm for 5 min. The supernatant was removed, and the precipitate was washed with ethanol and centrifuged at 5000 rpm, 4 °C for 15 min. Then, once again, the precipitate was washed with distilled water and centrifuged at 5000 rpm for 5 min. The obtained β-glucan compounds were dried in an air oven at 55 °C for 6 h.

#### 2.2.2. Physico-Chemical Parameters of Winery Yeast Lees

Total ash was determined in wine lees samples according to the method described by the International Organization of Vine and Wine OIV-MA-VI-07: R2000 [23], and the dry substance was determined by the simple method of removing water by heating to a temperature of 100–110 °C and determining the mass difference between the initial and final sample. The total carbohydrates in wine lees were determined using the Antron method. The absorption was recorded at 620 nm [24]. Lipids were determined according to the Bligh and Dyer method using a mixture of ethanol, chloroform and acetic acid [25]. The total protein content of the sample was determined using the Lowry method, which is a spectrophotometric assay that detects the presence of peptide bonds [26].

#### 2.2.3. Total Phenolic Compounds

The total content of polyphenols in yeast lees samples was determined by the Folin–Ciocalteu colorimetric method described by Makkar et al., 2003 [27]. The sample solution (0.1 mL) was mixed with 0.5 mL of Folin–Ciocalteu reagent and 0.4 mL of 7.5% sodium carbonate, and the absorbance was measured at 765 nm after 10 min at 37 °C. The total polyphenol content was expressed as mg gallic acid equivalents (GAE)/kg wine lees. The calibration curve of the polyphenols was performed by using gallic acid at concentrations of 10–200 mg/L with the regression coefficient R^2^ = 0.99872 and equation y = 0.00949× + 0.02950.

#### 2.2.4. Anthocyanin Content

The anthocyanin content was measured spectrophotometrically at 535 nm, following extraction with a water–ethanol solution [28]. Spectrophotometric assays were performed on a UV-VIS Shimadzu spectrophotometer (Shimadzu Corporation, Kyoto, Japan).

$$\mathrm{Total}\;\mathrm{anthocyanins}\;(\mathrm{mg}\;\mathrm{cyanidin}\;\mathrm{per}\;\mathrm{gram})=\frac{Absorbance\times dilution\;factor}{98.2}$$

where 98.2 is the extinction coefficient od cyanidin 3-galactoside.

#### 2.2.5. Mineral Content

The mineral content was determined using inductively coupled plasma optical emission spectrometry (ICP-OES) with a Thermo Scientific iCAP 6200 Duo spectrometer (Thermo Fisher Scientific, Cambridge, UK) controlled by the software iTEVA (Trial version) [29]. Ash obtained from the calcination was dissolved using high-purity reagents (HNO_3_, Merck, Germany, Suprapur grade).

#### 2.2.6. Microscopic Analyses of Winery Yeast Lees

The yeast sediments from the four types of wine were analyzed using a Zeiss Primostar 3 Stereomicroscope with AxioCam 208 Color Camera (Carl Zeiss IMT Co., Ltd., Shanghai, China) and built-in software, Zen, ver. 3.4.91.00000, at 100× magnification. The wine yeast sediment was subjected to a dilution of 50 relative to the dry substance and stained with methylene blue for the viability test and to allow the observation of cell morphology [30].

#### 2.2.7. Calculation of Extracted β-Glucan Yield

The yield of glucan compounds was determined gravimetrically, as the ratio between the weight of the obtained dry extract and the initial amount of residual yeast reported to the dry matter [30].

#### 2.2.8. Fourier Transform Infrared Spectroscopy (FTIR) of Extracted β-Glucans

The analysis was performed on the raw, undried samples of β-glucans. The determinations were performed using a Nicolet iS10 spectrometer from Thermo Scientific (Karlsruhe, Dieselstraβe, Germany), equipped with a diamond crystal. Measurements were performed in reflective absorbance mode (ATR-FTIR), at 4 cm^−1^ resolution in the range of a mid-infrared region of 650–4000 cm^−1^, with 32 scans in transmission mode at a resolution of 4 cm^−1^.

#### 2.2.9. Rheological Properties of β-Glucan Suspensions

To analyze the rheological properties of β-glucan, 2% solutions/suspensions of extracted β-glucan were prepared. The measurements were carried out on the first, second and seventh day of storage of the suspensions at a temperature of 4 °C. Before taking measurements, the suspensions were allowed to warm up to room temperature. For rheological testing, a MARS 40 rheometer (Thermo-HAAKE Co., Ltd., Karlsruhe, Germany) with parallel plate geometry (40 mm diameter) was used. The temperature was controlled at 20 °C. The shear rate was linearly increased from 0 to 1400 1/s [31].

#### 2.2.10. Statistical Analysis

All measurements were performed in triplicate. Data were analyzed by ANOVA and Tukey’s test (n = 3, confidence level 95%) by using the SPSS 26.0 (trial version) software (IBM, New York, NY, USA).

## 3. Results and Discussion

In this study, we used two extraction methods (alkali–acid and autolysis combined with ultrasound) and compared whether the extraction method had a greater influence on the yield and quality of β-glucans or the type of residual yeasts used. The macromolecular structures of the yeast cell wall (proteins, polysaccharides, lipids) present potential binding sites for various organic and inorganic molecules, present in must and wine, which makes the extraction of β-glucans difficult [18,32].

All four types of residual yeasts, in addition to the fact that they were obtained from wines of different varieties, also underwent different technological processes. The first type of yeast biomass came from a semi-dry white wine, produced from the Muscat grape variety. This type of wine contains sugars after the end of fermentation, of approximately 4 to 12 g/L of residual sugar. The activity of the winery yeast lees in this wine was stopped by the anaerobic conditions, the temperature and the presence of sulfites. The second type of residual yeast biomass was from a red wine, obtained from the Shiraz grape variety. It is recognized as one of the varieties with the highest polyphenol, tannins and antioxidant content [33]. The third type of residual yeast biomass contained yeasts collected after fermentation, but kept for half a year under refrigerated conditions. These residual yeasts were from the sweet wine produced from frozen grapes Muscat and Traminer, with a residual sugar content of over 45 g/L. The fourth type of residual yeast biomass was from the production of sparkling wine obtained by the classic fermentation method from Chardonnay, Pinot Noir and Pinot Blanc wine varieties. The specificity of these residual yeasts is that they are collected after disgorging stages, i.e., the yeast residue from the bottle is frozen and removed from the bottle.

Compared to the residual yeasts after alcoholic fermentation, all four types of these yeasts were in the inactive phase. In this phase, the *Saccharomyces cerevisiae* yeast cell undergoes a series of changes that allow it to survive in unfavorable conditions. This phase is essential for yeast survival in environments where there are insufficient nutrients or where conditions are otherwise hostile to active metabolic activity. One of the important changes that takes place is the thickening of the cell wall.

### 3.1. Physico-Chemical Characteristics and Mineral Content of Wine Lees

In order to understand the subsequent behavior and properties of yeast sediments, we first analyzed their physico-chemical properties. Table 2 shows the results of the physico-chemical analyses performed on the four types of wine lees.

The results of the analyses show that the winery yeast lees were rich in proteins (from 41.12 ± 0.87 to 45.98 ± 0.23%) and phenolic substances (from 142.19 ± 0.77 to 3350.17 ± 2.42 mg GAE/kg for red wine). Total phenolic content and total anthocyanin content in grape skins vary among grape varieties, and wine grape varieties have been observed to have higher phenolic and anthocyanin content compared to table grape varieties [34,35]. Research has shown that large amounts of beneficial polyphenols are present in pomace extracts and that the type of grape used, agronomic practices and winemaking method all influence the quantity and quality of the extracts. At the same time, 28.3915 ± 7.0 mg/kg of polyphenols was identified from Vermentino pomace and 11.3163 ± 6.5 mg/kg from Malvasia pomace obtained from Italian wines [36].

We obtained practically similar values for dry matter, ash and carbohydrates for all types of yeast lees. It is known that total lipids and the composition of fatty acids vary at different ripening stages and depending on the grape variety. At the same time, the lipid content increases significantly due to prolonged skin/pomace contact. Furthermore, the extraction of lipids and fatty acids increases linearly with the concentration of ethanol and the contact time with pomace [37]. In our case, we obtained lipid values between 4.71 ± 0.21% and 11.09 ± 0.95%. According to the obtained results, the yeast sediments from winemaking could serve as a source of proteins, whose nutritional value is expressed by the high content of essential amino acids [38,39].

The results of the analyses showed that the yeast sediments contained macroelements: K from 59.1 ± 0.14 to 99.7 ± 0.10 mg/g dry weight; Na from 0.1 ± 0.41 to 0.8 ± 0.24 mg/g dry weight; Mg from 0.4 ± 0.16 to 1.4 ± 0.04 mg/g dry weight; P from 3.2 ± 0.04 to 15.1 ± 0.07 mg/g dry weight; Ca from 1.9 ± 0.19 to 6.9 ± 0.17 mg/g dry weight; and S from 1.1 ± 0.21 to 3.9 ± 0.31 mg/g dry weight. We observed that red wine stood out with the maximum amounts of the six elements. At the same time, minerals present in the soil and transferred to the grapes of *Vitis vinifera* L. “Cabernet Sauvignon” harvested in three Mexican vineyards influenced the amounts and types of bioactive compounds present in the wine. The phenolic content and, therefore, the organoleptic characteristics of the wine are related to the mineral composition and the origin of the viticultural soil [40]. Most often, the mineral content is correlated with the temperature of the growing season and the hours of sunshine, as well as with the appearance of the vineyard. Studies of Chablis wine from the wine region in northern Burgundy, France, suggest that the soils and geology are not a primary source of minerality in the wine. On the other hand, the heat and sunlight of the growing season are relevant for the mineral content of the wine [41]. Mineral elements, along with volatile components and metabolites, are the basis of the geographical origin of the wine and are of great importance, because falsification of the origin is quite common in the wine industry [42,43]. Research studies based on characteristic variables (mineral elements, volatile components and metabolites) have very good performance. This demonstrates the effectiveness of a multi-source data fusion strategy for validating the authenticity of Chinese wine [44,45].

### 3.2. Microscopy of Winery Yeast Lees

Since three of the four wine yeast sediment samples were collected after the wine had been stored, we also analyzed these sediments with the help of a microscope to observe if the yeasts were still active and if there were other types of microorganisms that had developed in the meantime. Because yeast lees are a semi-solid product with a paste-like consistency, the 1:50 dilution ratio was chosen to achieve an optimal concentration of yeast cells that allowed clear visualization and accurate analysis under the microscope.

**Figure 1:**
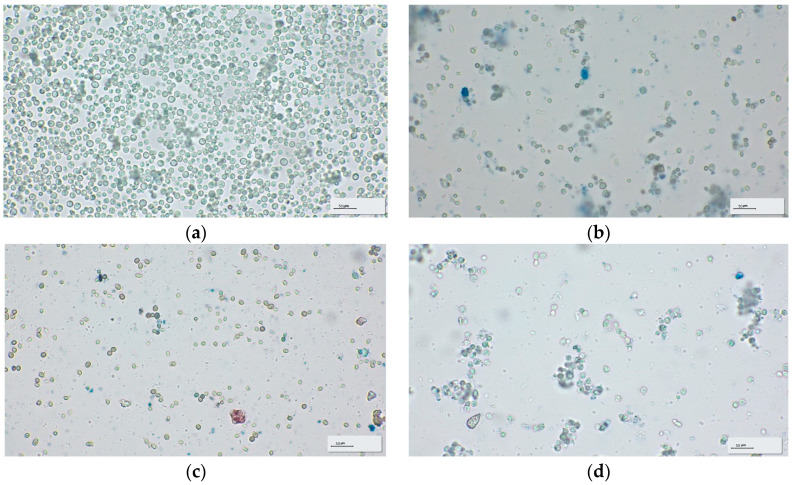
Microscopic images of winery yeast lees (100× magnification objective). (**a**) Semi-dry white wine (SVAM), (**b**) sweet white wine (SVR), (**c**) dry red wine (SVRS), (**d**) sparkling white wine (SVS).

The microscopic image of the semi-dry white wine (Figure 1a) shows a high concentration of yeast cells, most of which are intact, suggesting an incomplete or ongoing fermentation. This is typical of a semi-dry wine, where residual sugar levels are higher, supporting yeast activity. The image of the sweet white wine (Figure 1b) presents a smaller number of yeasts, many of them in degradation stages. Sweet wines are generally higher in alcohol and sugar, which can inhibit yeast activity and lead to cell death. The microscopic examination of dry red wine (Figure 1c) reveals that the number of yeast cells is even lower, many of them being fragmented or lysed. This is typical of dry wines, where fermentation is complete and the yeast is no longer active. In the case of white sparkling wine (Figure 1d), the image shows a significant presence of yeast cells, some intact, some decayed, suggesting yeast autolysis, a crucial process in the maturation of sparkling wines, where dead yeast contributes to flavor and texture development. Semi-dry white wine, having more residual sugar, keeps the yeast active for a longer period (Figure 1a). This results in a higher concentration of intact yeasts in the sediment, unlike dry or sparkling wines, where fermentation is complete and most of the yeasts have already been eliminated or autolyzed.

In the context of the above, we note that yeast sediments have a different microbiological characteristic that is primarily influenced by the technological process underlying the production of the four types of wines. More than that, the microbiological characteristic of the sediments (Figure 1a–d) confirms that the exploitation of microbial resources is important for improving the sustainability of the winemaking process. This is a fairly recent approach, and it seems that quite a few studies are being conducted on it so far. Reviewing the potential of microorganisms and their interactions as a natural, environmentally friendly tool, improving sustainability aspects throughout the production chain, including waste treatment, becomes imperative to ensure sustainability in viticulture [46,47].

At the same time, it is known that the microbiota of grapes depends on factors such as environmental characteristics, geographical location, cultivation mode and others [48,49,50]. However, there are three fundamental microorganisms in the winemaking process: *Saccharomyces cerevisiae* yeasts, *non-Saccharomyces* yeasts and lactic acid bacteria [49]. In fermentation, microorganisms metabolize the sugar present in the grapes and produce several metabolites responsible for the wine’s quality, including flavor and hue, among others [51,52]. In relation to wine production, it is known that during industrial fermentation, it is possible to inoculate microorganisms in order to improve the wine’s characteristics. On the other hand, the process of fermentation in artisanal conditions takes place naturally, and the microorganisms present come from the microbiota specific to the grapes [53,54,55,56].

### 3.3. Yield of Extracted β-Glucans

Table 3 shows the yield of β-glucans extracted by two methods. In both methods, ultrasound treatment was used to determine if it has an influence on β-glucan extraction. For both methods, ultrasound was used at frequencies of 25 kHz and 45 kHz. In the case of the acid–base method, three different concentrations of the NaOH solution (1, 1.5, 2 M) were tested.

The data obtained show that there are differences between the yield of β-glucans obtained by these two methods. The extraction of β-glucans using autolysis is much more efficient in terms of yield. For example, in semi-dry white wine, the yield with the autolysis-assisted method was 37.28–39.36%, while with the acid–base method, it was in the range of 2.77–19.76%. For sparkling white wine, the yield obtained by autolysis varied between 18.95 ± 0.49−41.34 ± 0.31% and that by acid–base extraction, between 5.11 ± 0.17 and 17.30 ± 0.30%. The action of cell enzymes was much stronger than the effect using hot NaOH, but the factor that remained to be elucidated was the purity of the β-glucans obtained by these two methods. At the same time, comparing application of the methods with and without ultrasound, there were no significant differences. The observed differences can be explained as being due to the bonds between mannoproteins and β-1,3 and β-1,6 structures at the cell wall level that have not been fully cleaved. Of course, these glucan-mannan structures have high potential for use as alternatives to antibiotics, and the future prospects underline the idea of using the two compounds. For example, for dry red wine, the extraction yield by autolysis varied with ultrasound, between 26.59 ± 0.0.6% and 30.91 ± 0.07%, and without it, measured 26.59 ± 0.04%. At the same time, for the same wine lees, the yield with acid–base extraction with ultrasound varied between 9.49 ± 0.29 and 18.98 ± 0.29, and without ultrasound, measured 13.80 ± 0.05%. We can say that the application of ultrasound in the case of these two methods in the given conditions did not have a significant influence. Also, the ultrasound frequency used did not influence the extraction yield in the case of both methods (e.g., for sweet white wine using the acid–base method, at 25 kHz the yield was between 5.99 ± 0.16% and 9.99 ± 0.30%, and at the frequency of 45 kHz, between 5.17 ± 0.19% and 6.70 ± 0.10%). The type of yeast used has the greatest influence on the yield of extracted β-glucans. The highest β-glucan yield using autolysis was observed in sparkling white wine (41.34 ± 0.31% without ultrasound), indicating that certain wine residues may respond more effectively to enzymatic degradation. The technological processes, the subsequent storage conditions of the wine and the way of keeping the winery yeast lees directly influence the extraction yield. The percentage of β-glucan obtained by the acid–base method was very close to that obtained by Karslioglu et al., 2021 [9], 3.47 ± 0.66% and 19.76 ± 0.58%, even though the author used yeast grown on culture medium. However, the percentage obtained by autolysis was much higher than the one reported by the same author. The yield obtained after extraction by autolysis was close to that obtained by Varelas et al., 2016 [8], 18.95 ± 0.49% and 39.36 ± 0.19%.

### 3.4. FTIR Spectroscopy

**Figure 2:**
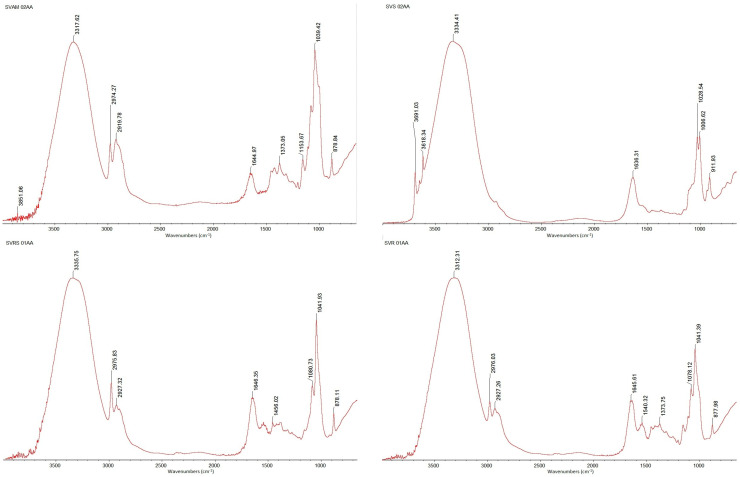
FT−IR−ATR spectra of the most representative samples.

The recorded FTIR spectra are shown in Figure 2. All samples have a few peaks that are repeated in each sample, namely in the range of 1040–1030 cm^−1^. The presence of absorption peaks in the 900–1200 cm^−1^ region confirms the C–C and C–O stretching vibrations, indicating that polysaccharides are a major component [11]. According to Binati et al. 2024, the peaks near 995, 1040 and 1025 cm^−1^ are typical for β-glucans (the peaks at 1025 are characteristic for β-1,4 glucans) [57]. In the sample SVS 02AA (sparkling wine yeast lees), there is also a peak near 1008 cm^−1^ that indicates the presence of β-1,6 glucans [58]. In the SVAM 02AA (semi-dry white wine) sample, we also observed a peak at 1153 cm^−1^, which corresponds to α-linked glucans. Another peak that could be found in practically all samples was at 1042, 1041 cm^−1^, indicating the presence of β-1,3-glucan [32]. Different peaks around 1636 cm^−1^ were mainly associated with the C=O stretching vibration and are characteristic for amide I, absorption of proteins [59]. In the spectra for samples SVAM 02AA and SVRS 01AA were found peaks at 878 cm^−1^; the absorption bands near 890 cm^−1^ are characteristic for β-anomeric configurations [60,61,62]. Another characteristic peak for a majority of the samples was found in the region 2950–2850 cm^− 1^, corresponding to C–H groups commonly found in polysaccharide [63,64]. A strong and broad band in the 3600–3000 cm^−1^ region (in our case, in all samples, it was a peak around 3300 cm^−1^) corresponded to the stretching vibration of abundant OH in polysaccharide [24,25,26,65,66,67,68].

Another peak that was found in several samples was near 2975 cm^−1^. According to author Hong et al., 2021, a well-resolved group of moderate bands located in the 3000–2500 cm^−1^ region can be assigned to the symmetric and asymmetric stretching vibrations of skeletal CH and CH_2_ in polysaccharides [67].

Infrared Fourier transform spectroscopy revealed that the examined samples contained a variety of glucan and polysaccharide types. The polysaccharide composition was confirmed by the primary absorption areas (900–1200 cm^−1^) that correspond to the C–C and C–O stretching vibrations. Furthermore, the diversity of glucan molecules was shown by the distinct existence of β-1,4, β-1,3, and β-1,6 glucans, as indicated by the various spectral peaks (particularly at 1000–1150 cm^−1^). A polysaccharide–protein-rich composition was suggested by specific protein-associated C=O vibrations (1636 cm^−1^) and broad bands in the 3300 cm^−1^ area, which are related to polysaccharide OH groups. The molecular fingerprints of the various glucan and polysaccharide types found in each sample, as observed in the specific spectral peaks, provided detailed insights into the structural diversity and composition of the samples. For instance, peaks associated with β-1,4, β-1,3, and β-1,6 glucans (1000–1150 cm^−1^) highlighted the variety of glucan linkages, while the C=O stretching vibration at 1636 cm^−1^ suggested the presence of protein–polysaccharide interactions. These spectral features confirmed the polysaccharide–protein-rich nature of the samples and underscored the complexity of their molecular composition [68].

### 3.5. Rheological Properties of β-Glucan Suspensions

**Figure 3:**
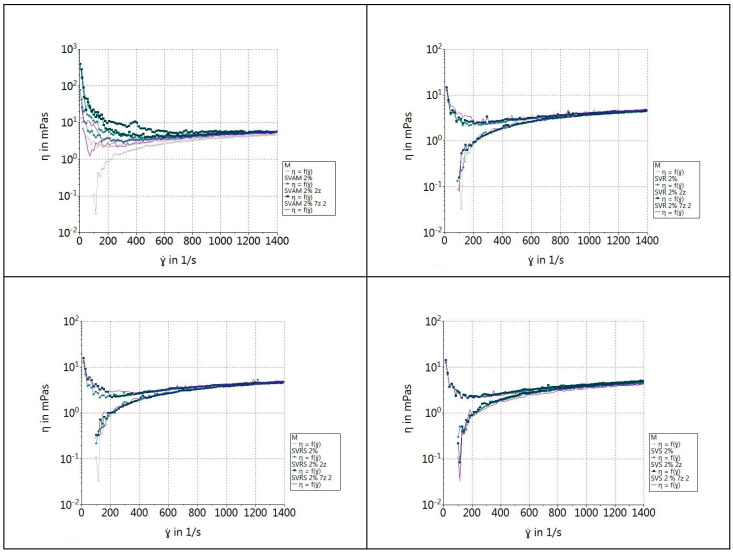
Shear rate dependence of viscosity for 2% suspension of β-glucans extracted by the acid–base method.

**Figure 4:**
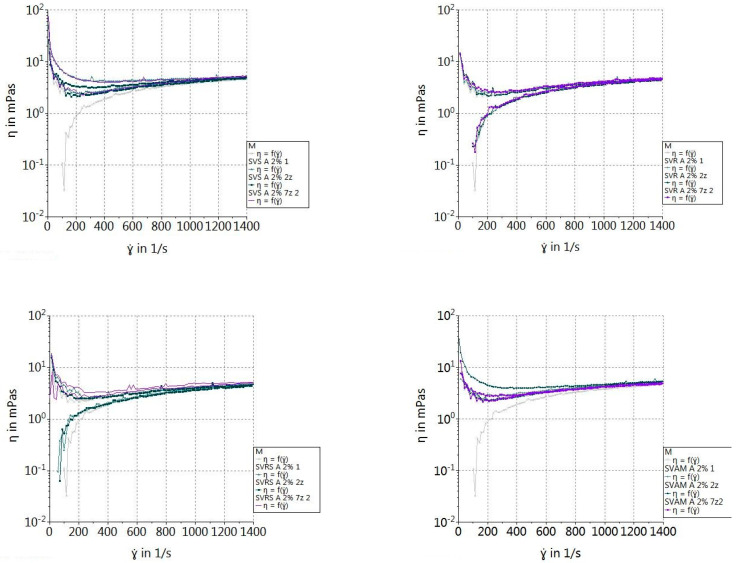
Shear rate dependence of viscosity for 2% suspension of β-glucans extracted by autolysis.

The results of these tests are presented in Figure 3 and Figure 4. In all the samples, we observed that the initial viscosity was increased, after which it stabilized with the increase in shear rates; this happened around the value of 200 1/s. This showed that the selected shear rate range was too large for this concentration. However, we could observe different behaviors of the four types of suspensions of β-glucan extracts. As the shear rate increased, the viscosity decreased significantly, indicating pseudoplastic or shear-thinning behavior. This phenomenon is common for suspensions containing polymers or supramolecular structures, where the applied forces break down the internal structure, thus reducing the viscosity. This is also mentioned by Lante et al. 2023 for solutions that have a concentration greater than 1% [69].

For both extraction methods, we could observe that the samples obtained from the yeast sediment of dry red wine (SVRS), sweet white wine (SVR) and sparkling white wine (SVS) had the same behavior and approached the control sample. However, the suspension obtained from yeasts from semi-dry white wine (SVAM) had a higher viscosity compared to the control sample in the entire time interval. The presence of a yield stress, which is often attributed to the presence of particles in the macromolecular medium, was indicated by an increase in apparent viscosity at low shear rate as the shear rate dropped. However, weak intermolecular interactions may also be responsible for this phenomenon. However, low-frequency viscoelastic measurements showed that the system had a propensity to exhibit gel-like characteristics. Physical cross-links created by intermolecular interactions could be the cause of this.

We can also state that there were no significant differences in viscosity between the suspensions obtained by autolysis and the acid-base method for the samples SVR and SVRS, and small differences for the samples SVAM and SVS. According to Petravi-Tominac et al. 2011, the differences in the β-1,3/1,6 ratio affect the functional properties of β-glucans [31]. The removal of β-1,6-glucan, which happens in harsh acidic conditions, as in the case of the two methods we used [12], leads to changes in the composition and chemical structure of β-glucan molecules and therefore induces changes in rheological properties [31].

## 4. Conclusions

The winery yeast lees represent a by-product rich in nutrients such as proteins, lipids, carbohydrates, mineral substances, and bioactive compounds such as polyphenols and β-glucans. The winery yeast lees analyzed had a protein content between 41.12 ± 0.87% and 45.98 ± 0.23%, lipid content between 4.71 ± 0.21% and 11.09 ± 0.95%, total phenolic content between 142.19 ± 0.77 mg GAE/kg and 3350.17±2.42 mg GAE/kg, and β-glucan content that varied between 2.77 ± 0.14% and 39.36 ± 0.19%. These results were due to the variability between the yeasts lees obtained by different winemaking technologies. Thus, the yield and the types of β-glucans and their properties are influenced by several factors, such as winemaking technology, the type of yeast used and the extraction method. In terms of yield, the extraction by autolysis was more efficient (between 18.95 ± 0.49% and 41.34 ± 0.31%) than the acid–base method (yield between 2.77 ± 0.14% and 17.30 ± 0.30%). Also, the type of yeast or winemaking technology has an important influence on the extraction of β-glucans from winery yeasts lees. Fourier transform infrared spectroscopy showed the presence of β-glucans in all extracted samples. The rheological properties of the 2% suspension also showed that both the extraction method and the winemaking technology have an influence on the viscosity of the β-glucans. Further studies on different types of yeast lees are necessary to improve the efficiency of utilizing these valuable compounds. At the same time, the obtained results on the extraction and properties of β-glucans contribute to a better understanding of how these compounds can be integrated into various functional foods, providing health benefits (β-glucans extracted from winery yeasts lees can be used to improve the texture and nutritional value of bread, fermented dairy products and natural juices).

## Figures and Tables

**Table 1:** Winery yeast lees and their provenance.

Winery Yeast Lees	Abbreviation	Grape Variety	Harvest Year	Manufacturer	Recovery Stage
Semi-dry white wine	SVAM	Muscat	2023	Chateau Vartely winery	Collected after the wine was stored
Dry red wine	SVRS	Shiraz	2023	Purcari winery	Collected after the wine was stored
Sweet white wine (Ice wine)	SVR	Traminer and Muscat	2023	Poiana winery	Collected after fermentation
White sparkling wine	SVS	Chardonnay, Pinot Blanc and Pinot Noir	2022	Purcari winery	Collected after disgorging the sparkling wine

**Table 2:** Physico-chemical characteristics and mineral content of wine lees.

Nr.	Parameters	Wine Lees			
		Semi-Dry White Wine (SVAM)	Sweet White Wine (SVR)	Dry Red Wine (SVRS)	White Sparkling Wine (SVS)
Physico-chemical characteristics					
1	Ash, %	0.04 ± 0.11 ^a^	0.03 ± 0.26 ^b^	0.04 ± 0.26 ^a^	0.04 ± 0.14 ^a^
2	Dry matter, %	24.16 ± 0.14 ^a^	21.09 ± 0.12 ^b^	26.09 ± 0.17 ^b^	12.22 ± 0.04 ^c^
3	Carbohydrates, % SU	21.16 ± 0.65 ^b^	24.61 ± 0.66 ^c^	19.15 ± 0.09 ^a^	20.31 ± 0.65 ^a^
4	Lipids, % SU	8.11 ± 0.49 ^a^	11.09 ± 0.95 ^b^	4.71 ± 0.21 ^c^	8.13 ± 0.96 ^a^
5	Proteins, % SU	41.12 ± 0.87 ^a^	32.62 ± 0.19 ^c^	45.98 ± 0.23 ^b^	42.42 ± 0.43 ^a^
6	Total phenolic substances, mg GAE/kg	162.12 ± 0.84 ^b^	192.59 ± 1.04 ^a^	3350.17 ± 2.42 ^c^	142.19 ± 0.77 ^b^
7	Total anthocyanins, mg GAE/kg fw	227.48 ± 1.14 ^a^	277.12 ± 0.94 ^a^	1311.08 ± 0.92 ^c^	269.98 ± 0.49 ^b^
Mineral content, mg/g					
1	K	59.1 ± 0.14 ^c^	67.9 ± 0.11 ^b^	99.7 ± 0.10 ^a^	93.2 ± 0.04 ^a^
2	Na	0.1 ± 0.41 ^b^	0.3 ± 0.04 ^b^	0.8 ± 0.24 ^c^	0.4 ± 0.11 ^a^
3	Mg	0.4 ± 0.16 ^b^	0.6 ± 0.23 ^c^	1.4 ± 0.04 ^a^	1.1 ± 0.21 ^a^
4	P	3.2 ± 0.04 ^b^	4.6 ± 0.29 ^a^	15.1 ± 0.07 ^c^	4.1 ± 0.43 ^a^
5	Ca	2.6 ± 0.11 ^a^	1.9 ± 0.19 ^b^	6.9 ± 0.17 ^c^	2.7 ± 0.07 ^a^
6	S	1.1 ± 0.21 ^a^	2.5 ± 0.41 ^b^	3.9 ± 031 ^c^	1.9 ± 0,15 ^a^

Mean values with different letters in the same column are significantly different (*p* < 0.05).

**Table 3:** Yield of the extracted β-glucan compounds, %.

Sample			Yield of β-Glucan Compounds, %	Sample		Yield of β-Glucan Compounds, %
Acid–base method assisted with ultrasound						
Semi-dry white wine	1 M NaOH, 25 kHz	SVAM 01AA	9.65 ± 0.30 ^a^	1 M NaOH, 45 kHz	SVAM 11AA	3.47 ± 0.66 ^b^
Dry red wine		SVRS 01AA	14.38 ± 0.45 ^a^		SVRS 11AA	16.39 ± 0.39 ^a^
Sweet white wine		SVR 01AA	5.99 ± 0.16 ^a^		SVR 11AA	5.17 ± 0.19 ^a^
Sparkling white wine		SVS 01AA	14.33 ± 0.22 ^b^		SVS 11AA	5.27 ± 0.25 ^a^
Semi-dry white wine	1.5 M NaOH, 25 kHz	SVAM 02AA	19.76 ± 0.58 ^a^	1.5 M NaOH, 45 kHz	SVAM 11AA	5.72 ± 0.44 ^b^
Dry red wine		SVRS 02AA	18.98 ± 0.29 ^b^		SVRS 11AA	9.49 ± 0.29 ^a^
Sweet white wine		SVR 02AA	9.99 ± 0.30 ^a^		SVR 11AA	6.70 ± 0.10 ^a^
Sparkling white wine		SVS 02AA	12.68 ± 0.12 ^b^		SVS 11AA	5.11 ± 0.17 ^c^
Semi-dry white wine	2 M NaOH, 25 kHz	SVAM 03AA	5.89 ± 0.56 ^a^	2 M NaOH, 45 kHz	SVAM 12AA	5.89 ± 0.33 ^a^
Dry red wine		SVRS 03AA	10.64 ± 0.37 ^a^		SVRS 12AA	11.36 ± 0.16 ^b^
Sweet white wine		SVR 03AA	9.65 ± 0.21 ^a^		SVR 12AA	6.23 ± 0.24 ^b^
Sparkling white wine		SVS 03AA	14.38 ± 0.15 ^a^		SVS 12AA	8.07 ± 0.08 ^c^
Autolysis assisted with ultrasound method						
Semi-dry white wine	Autolysis, 25 kHz	SVAM 01A	37.28 ± 0.29 ^a^	Autolysis, 45 kHz	SVAM 11A	39.36 ± 0.19 ^b^
Dry red wine		SVRS 01A	26.59 ± 0.0.6 ^a^		SVRS 11A	30.91 ± 0.07 ^b^
Sweet white wine		SVR 01A	21.97 ± 0.07 ^a^		SVR 11A	21.03 ± 0.20 ^a^
Sparkling white wine		SVS 01A	18.95 ± 0.49 ^a^		SVS 11A	37.06 ± 0.06 ^c^
Extraction without the use of ultrasound						
Semi-dry white wine		SVAM AA	2.77 ± 0.14 ^c^		SVAM 01A	37.62 ± 0.20 ^a^
Dry red wine		SVRS AA	13.80 ± 0.05 ^b^		SVRS 01A	26.59 ± 0.04 ^c^
Sweet white wine	2 M NaOH	SVR AA	6.46 ± 0.22 ^c^	Autolysis	SVR 01A	21.90 ± 0.17 ^a^
Sparkling white wine		SVS AA	17.30 ± 0.30 ^c^		SVS 01A	41.34 ± 0.31 ^a^

Mean values with different letters in the same column are significantly different (*p* < 0.05).

## Data Availability

The original contributions presented in the study are included in the article, further inquiries can be directed to the corresponding author.
